# Resource-Area-Dependence Analysis: Inferring animal resource needs from home-range and mapping data

**DOI:** 10.1371/journal.pone.0206354

**Published:** 2018-10-24

**Authors:** Robert E. Kenward, Eduardo M. Arraut, Peter A. Robertson, Sean S. Walls, Nicholas M. Casey, Nicholas J. Aebischer

**Affiliations:** 1 Centre for Ecology & Hydrology, Wallingford, Oxfordshire, United Kingdom; 2 Wildlife Conservation Research Unit, Department of Zoology, University of Oxford, Oxford, Oxfordshire, United Kingdom; 3 Department of Water Resources and Environment, Aeronautics Institute of Technology, São José dos Campos, São Paulo, Brazil; 4 Remote Sensing Division, National Institute for Space Research, São José dos Campos, São Paulo, Brazil; 5 Department of Plant Biology, State University of Campinas, São Paulo, São Paulo, Brazil; 6 Centre for Wildlife Management, School of Biology, Newcastle University, Newcastle upon Tyne, Tyne and Wear, United Kingdom; 7 Biotrack Ltd, Wareham, Dorset, United Kingdom; 8 Anatrack Ltd, Wareham, Dorset, United Kingdom; 9 Game and Wildlife Conservation Trust, Fordingbridge, Hampshire, United Kingdom; University of Kwazulu-Natal, SOUTH AFRICA

## Abstract

An animal’s home-range can be expected to encompass the resources it requires for surviving or reproducing. Thus, animals inhabiting a heterogeneous landscape, where resource patches vary in size, shape and distribution, will naturally have home-ranges of varied sizes, so that each home-range encompasses a minimum required amount of a resource. Home-range size can be estimated from telemetry data, and often key resources, or proxies for them such as the areas of important habitat types, can be mapped. We propose a new method, Resource-Area-Dependence Analysis (RADA), which uses a sample of tracked animals and a categorical map to i) infer in which map categories important resources are accessible, ii) within which home range cores they are found, and iii) estimate the mean minimum areas of these map categories required for such resource provision. We provide three examples of applying RADA to datasets of radio-tracked animals from southern England: 15 red squirrels *Sciurus vulgaris*, 17 gray squirrels *S*. *carolinensis* and 114 common buzzards *Buteo buteo*. The analyses showed that each red squirrel required a mean (95% CL) of 0.48 ha (0.24–-0.97) of pine wood within the outermost home-range, each gray squirrel needed 0.34 ha (0.11–1.12) ha of mature deciduous woodland and 0.035–0.046 ha of wheat, also within the outermost home-range, while each buzzard required 0.54 ha (0.35–0.82) of rough ground close to the home-range center and 14 ha (11–17) of meadow within an intermediate core, with 52% of them also relying on 0.41 ha (0.29–0.59) of suburban land near the home-range center. RADA thus provides a useful tool to infer key animal resource requirements during studies of animal movement and habitat use.

## Introduction

That an animal’s home range contains vital resources for survival and reproduction seems obvious, but identifying and quantifying resource requirements is less so. Early visual observations of bird territories showed that territory size could correlate inversely with food supply [1,2]. These observations were followed by a similar and growing body of relationships between range size and habitats from elusive species, for which locations could be collected systematically only by tracking, including raptors [3,4], large cats [5,6], squirrels [7], lagomorphs [8], deer [9–11], bears [12,13], and moose [14].

At the same time, other studies were considering how data from tracked animals could be used to show animal relative preferences for particular habitats or resources [15–31]—when habitats were considered, the implicit assumption was that they were a proxy for resources. Resource selection functions express habitat or resources that animals use in terms of those available to them. There are several difficulties with this approach, including (i) the unit-sum constraint, by which one strongly avoided habitat (e.g. water for a squirrel) tends to make all others seem preferred, (ii) pseudo-replication, if locations from the same animal are treated as independent observations, and (iii) the definition of what is available or (iv) used.

The first two difficulties were solved by treating (i) habitat use as a compositional problem, and (ii) animals rather than locations as sample units [22]. However, the delimitation of what is available [32,33] or used [34] is less tractable. Should one consider habitat availability in the whole of a study area, despite some areas being unoccupied? Or should one choose areas around each location or path of an animal [24,26], perhaps using behavioral mechanisms to define what is accessible from each point or trajectory [27]? Similarly, if an animal lives in a landscape where resource-bearing patches have a fragmented distribution, should one consider all habitats within a chosen home-range outline to be used by the animal?

Here we interpret resource use through its role in structuring animals’ home-ranges. The fundamental principle is that, in a heterogeneous landscape, the resources an animal needs occur in patches that vary in size, shape and distribution [35], and that in order to benefit from these resources each animal will adjust the size, shape and structure of its home-range so that it encompasses the minimum required amount of this resource. This agrees with predictions from optimal-foraging theory, which states that in searching for food animals will try to optimize the energy budget [36–39] and that resource dispersion will influence range size [2,40,41].

In this paper, we introduce Resource-Area-Dependence Analysis (RADA), a new method that uses a sample of tracked animals and a categorical map depicting resource distribution to infer where important resources are accessible and to estimate the minimum area required for such resource provision. We illustrate our technique by analyzing data from 15 red squirrels (*Sciurus vulgaris*), 17 gray squirrels (*S*. *carolinensis*) and 114 common buzzards (*Buteo buteo*). We use these results to infer the key resource requirements of each species and discuss the wider application of this approach to the study of animal movement and resource use.

## Materials and methods

The data were collected prior to 1994, during a period before local ethics committees were required to review non-invasive radio-tagging of wildlife, as stated in the Animals (Scientific Procedures) Act 1986: *“The ringing*, *tagging or marking of an animal*, *or the application of any other humane procedure for the sole purpose of enabling an animal to be identified*, *is not a regulated procedure if it causes only momentary pain or distress and no lasting harm*” (page 2, section 2.5). Nevertheless, the permissions for squirrels obtained from the then Nature Conservancy Council and Ministry of Agriculture, Fisheries and Food included review of the proposed marking on animal welfare grounds, as did those for marking buzzards from the British Trust for Ornithology, which already at that time had a committee dedicated to the ethics of marking. Elton Estates Ltd., British Petroleum Ltd. and many South Dorset landowners generously granted land access during the entire data collection process.

### RADA model

**Fig 1:**
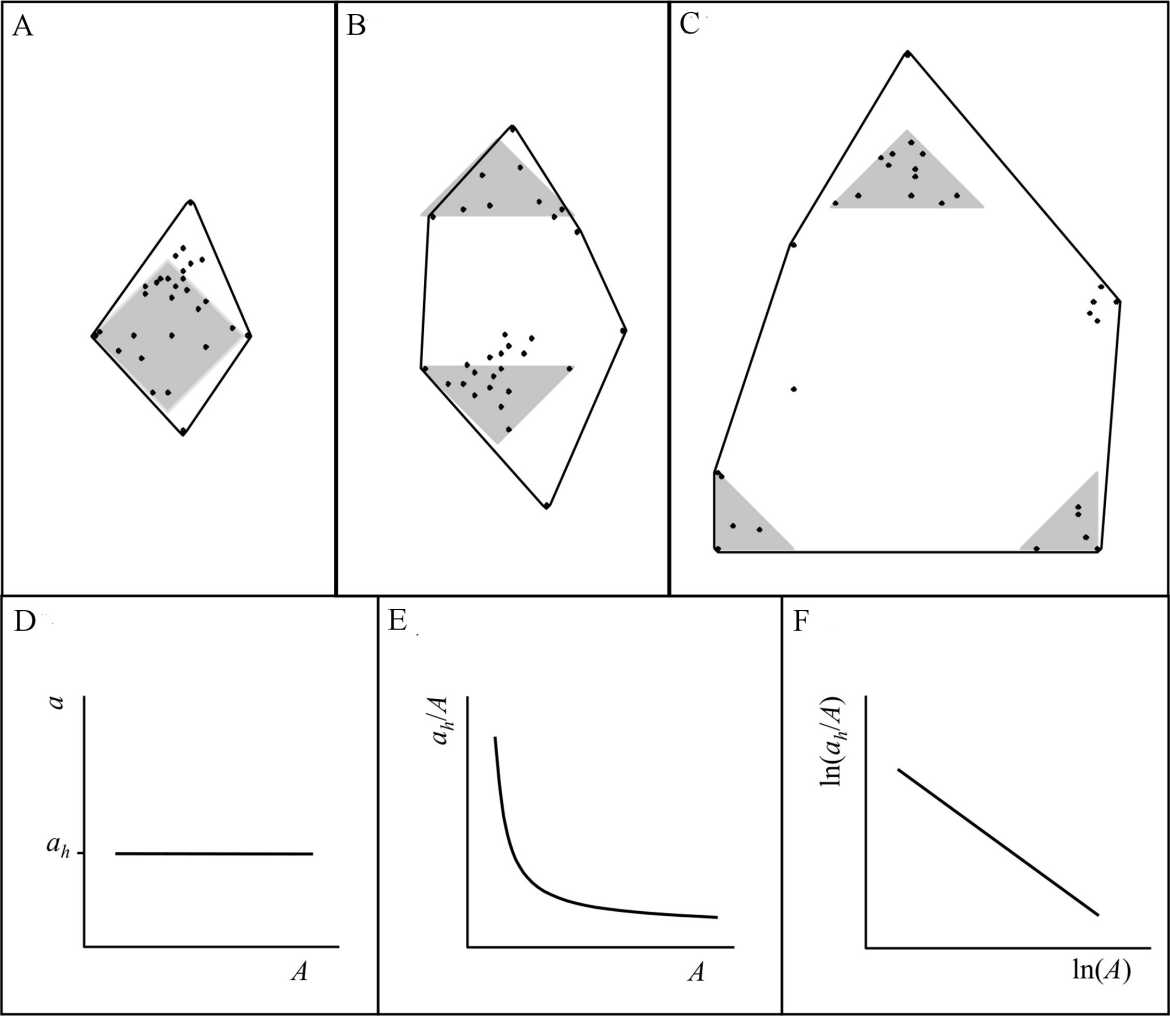
RADA model schematic diagram. 100% convex polygon peeled by distance from kernel range center (*Xk*_*100*_; for abbreviations used to denote home range estimators and cores, see S1 Appendix) of three animals requiring the same *a*_*h*_ (A) in one patch, (B) in two patches and (C) in three patches. For a population of animals that maintain an *a*_*h*_ in their home-ranges, a plot of *a*_*h*_ against *A* yields a horizontal line at y = *a*_*h*_ (D). (E) When *a*_*h*_*/A* is plotted against *A* the plot becomes hyperbolic, and (F) when both axes are ln-transformed it becomes linear with slope *b* = -1.

Consider a population of animals with home-ranges within a particular heterogeneous landscape and with similar minimum requirements for a certain resource *h* (we extend our approach to multiple resources later). Assume that the landscape is divided up into patches belonging to a range of categories, and that patch classification has been done in such a way that acquiring resource *h* is mainly associated with a single landscape category (e.g. based on prior ecological knowledge). Assume also that the amount of resource within a patch belonging to that category is, on average, proportional to patch size. In the absence of variation, the minimum requirement for the resource *h* translates into a minimum area *a*_*h*_ providing *h*, such that *a*_*h*_ is contained within each animal’s home-range. An animal for which *a*_*h*_ is available in one large patch will have a smaller home-range area (Fig 1A) than, for example, two other animals for which *h* is available in smaller disjunct patches that sum to *a*_*h*_ (Fig 1B and Fig 1C, see also [25]). At the population level, a plot of *a*_*h*_ against range area *A* yields a straight line parallel to the *x*-axis, i.e. with zero slope (Fig 1D, noting that *A* ≥ *a*_*h*_ if *a*_*h*_ is a minimum requirement; see also [25]). In contrast, an area *u* of a map category that is visited randomly within a home-range because it offers no resource will be increasingly found in large ranges as the animal moves between other, more valuable patches, so a plot of *u* against *A* will yield a positive slope.

In reality, there will be variation in *a*_*h*_ between animals, so as ecologists our aim is to identify *h* and find the average value of *a*_*h*_, which will be the best estimate of the minimum resource requirement at the population level. Intuitively, one would identify *h* from the horizontalness of the relationship of *a*_*h*_ versus *A* (Fig 1D), estimating *a*_*h*_ from extrapolation of the horizontal line to where it crosses the *y*-axis. However, an approach based on testing for a horizontal line is inappropriate, because the lack of a significant slope is the null hypothesis in a standard statistical regression. We consider instead the relationship between *a*_*h*_*/A* and *A*. Plotting *a*_*h*_/*A* against *A* yields a hyperbola (Fig 1E), and plotting the log-transformation of both variables yields a straight line (Fig 1F) described by ln(*a*_*h*_/*A*) = *b* ln(*A*) + *c*, with *b*<0. As ln(*a*_*h*_/*A*) = ln(*a*_*h*_)–ln(*A*), ln(*a*_*h*_)–ln(*A*) = *b* ln(*A*) + *c*. Since one expects the minimum required area to be estimated when *a*_*h*_ = *A*, i.e. ln(*a*_*h*_/*A*) = 0, solving for this case yields ln(*a*_*h*_) = –*c/b* (which is the *x*-axis intercept) and *a*_*h*_ = exp(-*c*/*b*). With a perfect hyperbola, *b* = -1, and *a*_*h*_ = exp(*c*). Similar reasoning applies to Pearson’s correlation coefficient *r* between ln(*a*_*h*_/*A*) and ln(*A*): *r* is expected to be negative, and takes a value of -1 for a perfect hyperbola.

From a statistical viewpoint, the ecologist samples the population of animals by marking a number *I* of individuals assumed to be representative. Each animal *i* will have a measured home-range area *A*_*i*_ and an area *a*_*h*,*i*_ within it that provides resource *h*. The reasoning made at the population level suggests the possibility of using the *A*_*i*_ and *a*_*h*,*i*_ to identify *h* and estimate *a*_*h*_. When dealing with real data, however, deviations from the theoretical situation above will result in a point dispersion such that *b* ≠ -1 and *r* > -1. Deviations from a perfect hyperbola may arise from *h* not being homogeneously accessible, inaccurate mapping or individual variation in movement costs or *h* requirements.

Think of a white rhino *Ceratotherium simum*. The male is a solitary grazer that safeguards patches of grassland within its territory [42]. One such male might find all the food it needs within a grassland patch that is so rich it could even feed two rhinos. However, as the patch is within its territory, no other rhino will explore it while it is the resident. One day, a larger white rhino arrives and after an intense fight expels the previously resident one, taking hold of the rich patch. This larger animal will use more of what the patch has to offer, but, back to the model, *a*_*h*_ would be the same for both rhinos. The effect on *a*_*h*_ of individual variability in *h* requirement will have been compensated by variability in *a*_*h*_ richness. By analogy, this also applies to the variation in movement cost, e.g. the same rich patch will equally feed a young adult or a large, dominant male that often fights for females and thus has a much higher movement cost.

In RADA, the net effect on *A* or *a*_*h*_ of these unquantifiable variables is treated as uncertainty, which pushes *b* and *r* away from -1 and is a source of conservative bias. The way to minimize it is to collect a large enough sample of individuals and estimate their home-ranges well, while preparing the map carefully and defining the statistical population being analyzed in a way that ensures as much homogeneity as possible (e.g. by considering adults and immatures separately).

### Complications and solutions

Complications may arise owing to (i) masking among map categories, ii) lack of independence between *a*_*h*_/*A* and *A* and (iii) a subset of the home-range cores lacking a particular resource-containing map category.

**Fig 2:**
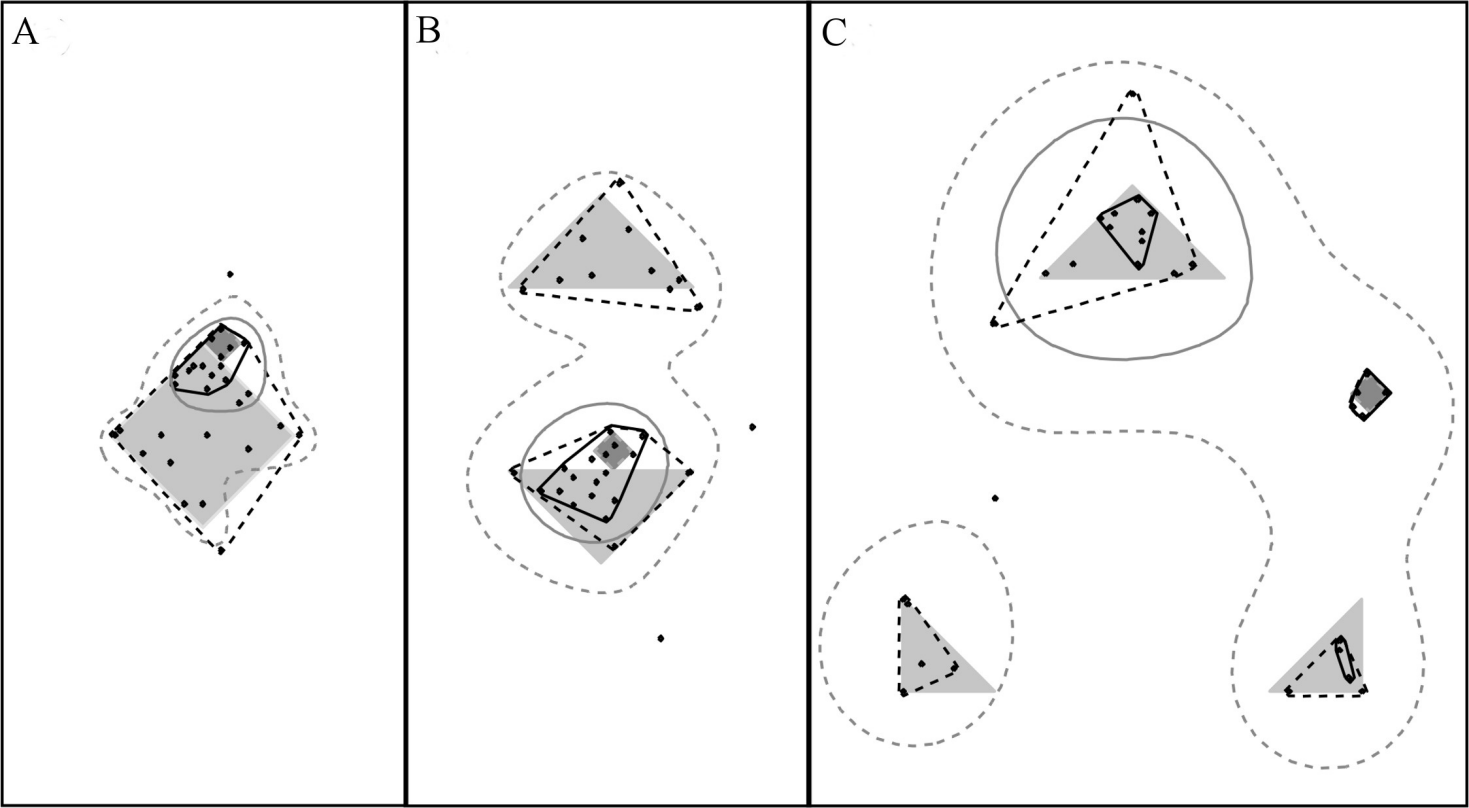
Presence within *A* of an abundant resource-providing map category may mask result for another less abundant one (solution to this issue explained in body of text). Panels A, B and C show, respectively, the ranges of the three figurative animals in Fig 1A, Fig 1B and Fig 1C. When a second resource-providing map category (dark gray) with, say, 1/20 the volume of the main resource-providing map category (pale gray) is present, the former may be entirely excluded from small home-range cores. RADA solves this issue by means of a stepwise regression procedure. Shown here are *K10d*_*95*_ (dotted gray), *K10d*_*50*_ (solid gray), *Cx*_*95*_ (dotted black) and *Cx*_*50*_ (solid black).

The first problem–masking among map categories—arises when animals in a sample each require *a*_*h*_ of *h*, but also a much smaller area *a*_*g*_ of resource *g*–the role of *g* may be concealed (Fig 2) owing to the issue affecting proportions known as the ‘unit-sum constraint’ [22,43]. We solved this by following a conditional probability approach [44], which involved subtracting *a*_*h*_ from *A*_*i*_ when considering each of the *J-*1 remaining map categories and excluding cases when _-*h*_*A*_*i*_ = 0 (where−_*h*_*A*_*i*_ = *A*_*i*_*−a*_*h*,*i*_).

The second problem–lack of independence between *a*_*h*_/*A* and *A–*occurs because *A* is present on both sides of the regression: ln(*a*_*h*_/*A*) = *b* ln(*A*) + *c*. A consequence of this is that zero is no longer the expected value under the null hypothesis, and assessing departure of *b* and *r* from zero with parametric statistics would lead to detecting spurious relationships. We therefore opted for testing their significance against the null hypothesis of no relationship using randomization. The randomized empirical distributions include the effect of the dependence, so the statistical tests based on the randomizations automatically take any such dependence effects into account. With 999 randomizations, an observed value was significantly different from the random values, in a two-tailed test, if it was less than 50 (for *P* <0.10), 25 (for *P* <0.05) or 5 (for *P* <0.01) from the top or bottom edges of the distribution of random values, or had no random value beyond it (for *P* ≤ 0.002 from one run or *P* ≤0.001 in both of two runs). Using Ranges 9 [45], we estimated 999 values for *I* outlines selected at random with replacement from the *I* ranges in the data set, and then randomly placed (rotated and displaced) on the map within a convex hull encompassing the most expansive outline of the relevant estimation algorithm for all *I* ranges.

The third problem–subset of *A* lacking a particular resource-containing map category–was addressed with two different approaches: (i) omitting these ranges from the analysis, or (ii) using the transform log(*β* + *a*_*j*,*i*,_/*A*_*i*_), where *β* = 0.01 for map categories with mean proportions greater than 0.10 and *β* = 0.001 for less frequent categories [22].

### Implementation

For the development of this technique, we tested *K* (= 25) range estimator variants and *m* (= 1 or = 15) core sizes. Let us denote the *A*_*i*_ estimated using a particular *K* and *m* by *A*_*i*,*k*,*m*_, and the *a*_*h*_ contained in *A*_*i*,*k*,*m*_ by *a*_*h*,*i*,*k*,*m*_. For each regression between ln(*A*_*i*,*k*,*m*_) and ln(*a*_*h*,*i*,*k*,*m*_/ *A*_*i*,*k*,*m*_), we recorded the observed value of Pearson’s correlation coefficient *r*, the slope *b*, the standard error of *b*, the area intercept *c* (on a logarithmic scale) and the percentage of ranges with none of the map category in the core. We used *r* to assess the strength of the relationship and compare with random, and divergence of *b* from -1 to indicate variability in resource accessibility or in individual behavior or resource requirement.

Statistics from randomization included the mean and median values for *r*, *z* for the difference of mean *r* from the observed *r*, with associated 95% confidence limits (on the assumption that *r* is distributed normally), the number of random *r* values more extremely negative than the observed value, mean values for *b*, its SE and *c* by randomization, and, finally, the proportion lacking a map category at first ‘throw’ was recorded and the means ± standard errors were calculated (this proportion at ‘first throw’ allowed testing, using a binomial distribution, whether non-random inclusion of map categories in observed outlines indicated resource-based placement of the ranges). Geometric means, and other estimates back-transformed from logarithms, are shown with 95% confidence limits.

We began the analysis by visually inspecting the log-log plots (as in Fig 1F), searching, in particular, for signs of heteroscedasticity. Then, for *J* > 2 map categories, in step 2 we identified the map category presenting the strongest significant negative correlation with range area; if two yielded similar negative correlations, we considered the most prevalent category (owing to possible masking of the less prevalent by the latter, as explained above). For step 3, we addressed the masking problem by subtracting *a*_*h*,*k*,*m*,*i*_ from *A*_*k*,*m*,*i*_ when considering each of the *J-*1 remaining map categories, excluding cases of _-*h*_*A*_*k*,*m*,*i*_ = 0 (where−_*h*_*A*_*k*,*m*,*i*_ = *A*_*k*,*m*,*i*_*−a*_*h*,*k*,*m*,*i*_). We sought negative relationships between ln(*a*_*k*,*m*,*i*,*j*_/_*-h*_*A*_*k*,*m*,*i*_) and ln(_*h*_*A*_*k*,*m*,*i*_), a conditional probability approach [44]. The process was repeated by removal of further resource-containing map categories in a stepwise fashion, step four being examination of ln(*a*_*k*,*m*,*i*,*j*_ /_*-h*,*-g*_ *A*_*k*,*m*,*i*_) and ln(_*-h*,*-g*_ *A*_*k*,*m*,*i*_) where _*-h*,*-g*_ *A*_*k*,*m*,*i*_ = *A*_*k*,*m*,*i*_*−a*_*h*,*k*,*m*,*i*_−*a*_*g*,*k*,*m*,*i*_, for the *J*-2 map categories differing from those containing *h* and *g*. We repeated map category removal until no negative *r* value was significant (for a flowchart of the steps involved in this analysis, see Discussion).

To assess possible departures from the model’s assumptions, we noted whether observed *b*_*j*,*k*,*m*_ differed significantly from -1. If that was the case, we considered the mean proportion of *a*_*h*_ (i.e. ${\bar{a}}_{h}$) a more realistic estimator of habitat requirement than the minimum estimated via extrapolation of regression. In Ranges 9, RADA was implemented such that analyses involving several *A*_*k*,*m*_ could be run simultaneously [45].

### Three examples: Red and gray squirrels and common buzzards

We applied RADA to red squirrels, gray squirrels and common buzzards in southern Britain. The data were collected prior to 1994, during a period before local ethics committees were required to review non-invasive radio-tagging of wildlife, as stated in the Animals (Scientific Procedures) Act 1986: *“The ringing*, *tagging or marking of an animal*, *or the application of any other humane procedure for the sole purpose of enabling an animal to be identified*, *is not a regulated procedure if it causes only momentary pain or distress and no lasting harm*” (page 2, section 2.5). Nevertheless, the permissions for squirrels obtained from the then Nature Conservancy Council and Ministry of Agriculture, Fisheries and Food included review of the proposed marking on animal welfare grounds, as did those for marking buzzards from the British Trust for Ornithology, which already at that time had a committee dedicated to the ethics of marking. Elton Estates Ltd., British Petroleum Ltd. and many South Dorset landowners generously granted land access during the entire data collection process. The data files for red and gray squirrels, and the radio-tracking data for buzzards, are available for public use through the Dryad Digital Repository via the following link: http://dx.doi.org/10.5061/dryad.8n183. The Land Cover Map of Great Britain, which was used in the analysis involving buzzards, is available against permission at public site http://www.ceh.ac.uk/services/land-cover-map-1990.

The 15 red squirrels were the 14 used by [46] to correlate body mass with range size, plus one that was omitted from the previous analysis as its weight was not recorded. Convoluted and fragmented woodland (5 ha) in 17 patches, set in grassland around oil-production sites on the 13-ha Furzey Island in Poole Harbour, were digitized as vectors from an aerial photograph (see Fig 1 in [46]). The woodland was dominated by Scots pines (*Pinus sylvestris*) and the demography of the squirrels depended on the crops of pine cones [47].

The 17 gray squirrels were those radio-tracked during July 1989 at Elton estate [22], data for plotting also in [45]. The map was created with six categories, digitized as (1) two blocks of mature deciduous woodland (dominated by oak *Quercus robur*) totaling 27.9 ha, (2) a 1.5 ha patch of mature larch (*Larix decidua*), (3) a 6.3 ha plantation of young beech (*Fagus sylvatica*), (4) a 4.0 ha *Thuja* plantation and (5) surrounding fields of wheat extending for more than 75 ha. The squirrels fed mainly on acorns in autumn and winter, with tree flowers and larch cones taken in spring, and also ate ripening wheat outside the 40 ha of woodland while being tracked [48].

The 114 common buzzards were monitored in Dorset during October 1990–94, shortly after their main autumn dispersal period [49], when they were feeding mainly on invertebrate prey. For these wide-ranging raptors, mapping data were provided as 25x25-m pixels in the Land Cover Map of Great Britain (LCMGB), which was developed by supervised likelihood classifications of combined winter and summer Landsat Thematic Mapper scenes imaged in November 1989 and July 1990 [50,51]. During analysis, the 25 land-cover types were initially grouped into 16 map categories: (1) sea and inland water, (2) coastal zones (including beach mudflat and saltmarsh), (3) sparse grassland of dunes and heaths, (4) short (grazed and mown) grass, (5) seasonally long grass (mainly meadows), (6) marshland, (7) open shrub areas of mainly heath, grass and bogs, (8) dense shrub areas, (9) bracken (*Pteridium aquilinum*), (10) scrub and orchards (11) broadleaf or mixed woodland, (12) conifer woods, (13) arable crops, (14) suburban, (15) urban land and (16) bare ground including felled woodland. Inland map categories were relatively fine-grained with respect to buzzard ranges (see Fig 1 in [52]). RADA, thus, was applied to the following map categories (% of total 160,000 ha map area): sparse grass (2.5%), short grass (18.0%), seasonally long grass (34.5%), arable crops (14.9%), deciduous and mixed woodland (14.0%), conifer woodland (4.9%), suburban land (3.2%), sparse shrub (2.0%), dense shrub (1.6%), scrub and orchards (1.5%) and marshland (1.0%).

For all three species, home-range core estimates were based on standard samples of 30 locations, recorded by triangulation during VHF radio-tracking in morning, mid-day and afternoon (intervals which gave good temporal independence in autocorrelation analyses—for 10 days across two consecutive weeks. These sample sizes gave stable seasonal home-range core areas when plotted as density-based isopleths and MCPs [53], although larger samples might have had less method-dependent variance for the most complex outlines, of polygons based on restricted edges and nearest-neighbor cluster analysis [54].

## Results

### Red squirrels

**Fig 3:**
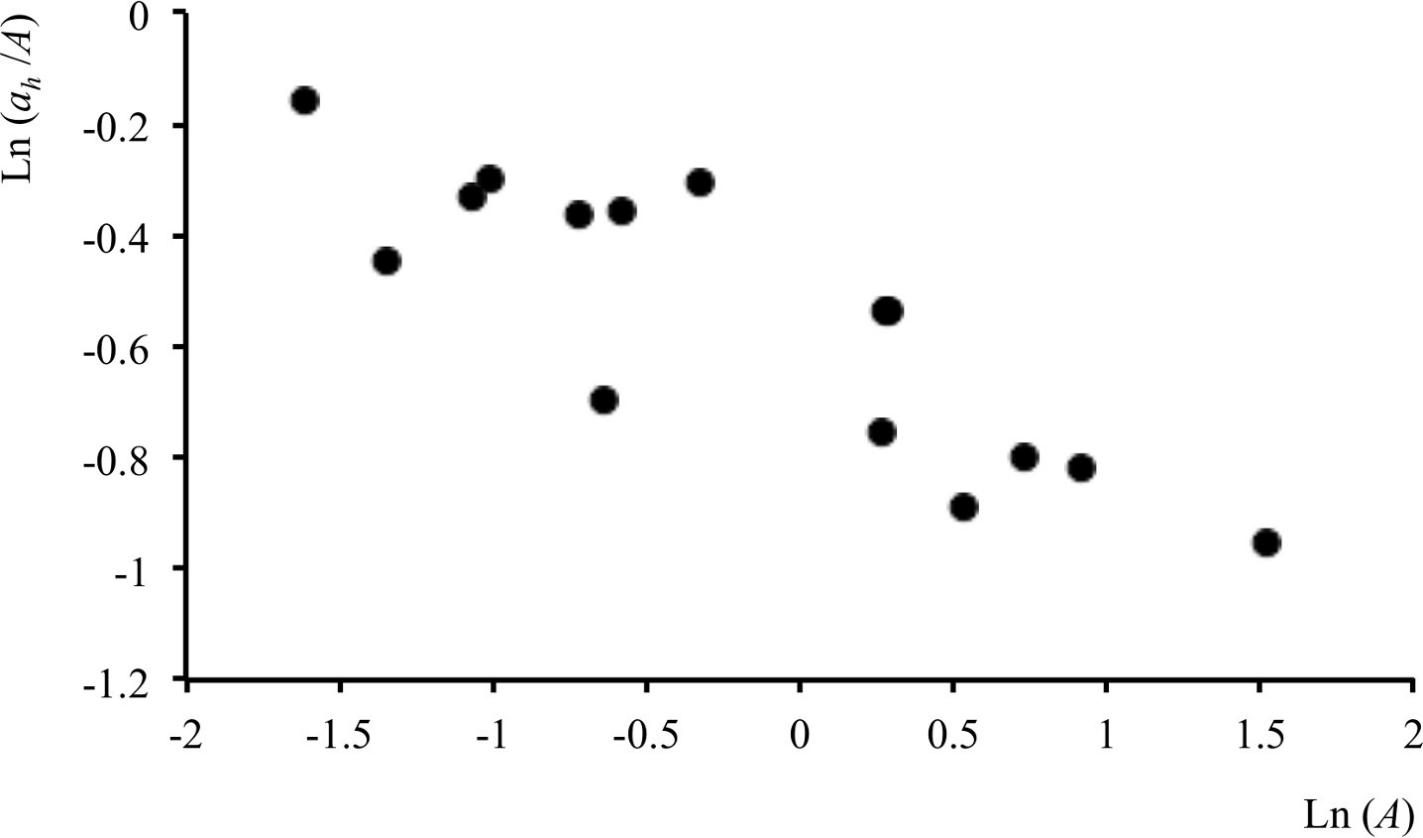
RADA results for red squirrels and pine woodland. Proportion of pine woodland (*a*_*h*_ /*A*) as a function of home-range area (*A*). *A* estimated using *ix*001.

Red squirrels on Furzey Island had their resources distributed in fragmented pine woodland in little-used grassland around tarmac areas, the whole being surrounded by sea. For representing the individuals’ home range cores, the more expansive, location-density algorithms (ellipses and kernel contours) tended to include tarmac or sea, both of which are seldom used by squirrels. An equally strong RADA result was obtained for tighter outlines round *ix001* clusters that contained on average 94% of the locations, which thus seemed a better representation of their home ranges. Indeed, this showed the strongest and most significant correlation with the proportion of pine woodland (S1 Table, Fig 3): observed *r* = -0.86 (n = 15), randomized mean *r =* +0.06 (95% CL = -0.54, +0.59, n = 999), with no random value below -0.86 (*P* ≤ 0.002). Prediction for mean minimum required woodland area was then 0.08 ha (0.01–0.17). That the slope of the regression, *b* = -0.23 (±0.04), was considerably shallower than -1 (*P* <0.001) suggested that red squirrels were not exploiting resources uniformly—individuals that had the smallest ranges either had the best of the pine woodland, used it most efficiently or required less resource as they moved less. Geometric mean woodland area (${\bar{a}}_{h}$) was 0.48 ha (0.24–0.97) in the *ix001* polygons.

### Gray squirrels

The home ranges of gray squirrels within the rectangular landscape patches were, as for red squirrels, also better represented by tighter outlines. With concave polygons round clusters that included 90% of locations, *Cv*_90_, and prior to excluding any map category, the correlation with mature deciduous woodland was *r* = -0.54 (n = 17), mean random *r* = +0.06 (95% CL -0.47, +0.52, n = 999) with 6 values less than -0.54 (*P* < 0.05). Within *Cv*_90_ outlines, mature deciduous woodland (mean = 38%) was considerably more common than wheat (mean = 5.3%), meaning that the former could mask possible relationships for the latter. To avoid this, the analysis was also run before (for comparison) and after omitting mature deciduous woodland. Omitting it improved results for wheat from *r* = -0.56 (n = 17, *P* < 0.01 by randomization) to *r* = -0.73 (n = 17, *P* < 0.002). From this, predicted mean minimum wheat requirement was 0.035 ha. A relatively similar mean estimate of 0.046 ha within *Cv*_90_ outlines reflected a regression slope close to -1, reinforcing the strong pattern of wheat-area-dependence. The squirrels were not usually recorded more than 10 m from the edge of the wood and usually fed along a strip of 30–50 m of wheat field.

**Fig 4:**
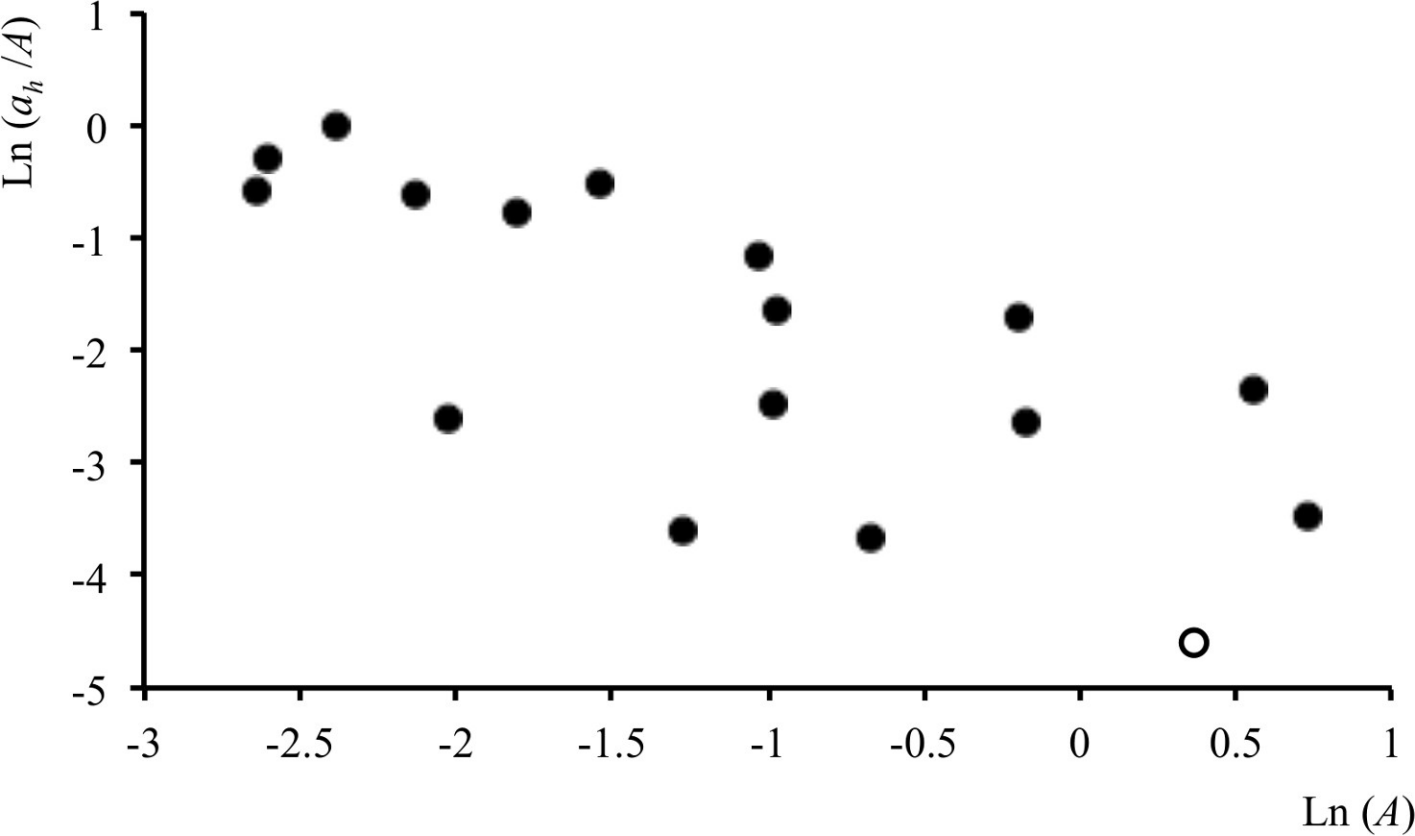
RADA results for gray squirrels and wheat. Proportion of wheat (*a*_*h*_ /*A*) as a function of home-range area (*A*) after excluding woodland; ‘ represents a squirrel lacking wheat in its range. *A* estimated using *Cv*_90_.

In contrast to what happened in range outlines, within the study area as a whole wheat was more common (48.6 ha) than woodland (18.1 ha). This led, during randomization, to large outlines tending to contain more wheat, which could lead to masking of woodland. To account for this, the analysis was also run excluding wheat and two squirrels that had no mature deciduous woodland within *Cv*_90_ outlines (Fig 4). Significance for woodland increased from *r* = -0.54 (n = 15, *P* < 0.05 by randomization) to *r* = -0.56 (n = 15, *P ≤* 0.005) with mean random *r* = +0.38 (95% CL -0.09, +0.67). There was 0.34 ha (0.11–1.12) of mature deciduous woodland in the 0.89 ha (0.47–1.68) *Cv*_90_ polygons.

### Common buzzards

The strongest correlations before removal of any map category were for seasonally long grass within *Koad* outlines (adaptive kernel density plots with least-squares optimizing). *Koad*_99_ gave the most significant observed *r* = -0.20 (n = 114), differing from random *r* = +0.10 (95% CL -.07, +0.27, n = 999) with all values > -0.20 in two randomization runs (*P <* 0.001). However, kernel plots that include 99% of location density distributions are extremely expansive outlines, likely to cover large areas not used by the birds. Moreover, the slope was very shallow (*b* = -0.06), so that the prediction from the intercept for meadow was close to 0 and its upper 95%CL corresponded to only 0.05 ha.

**Fig 5:**
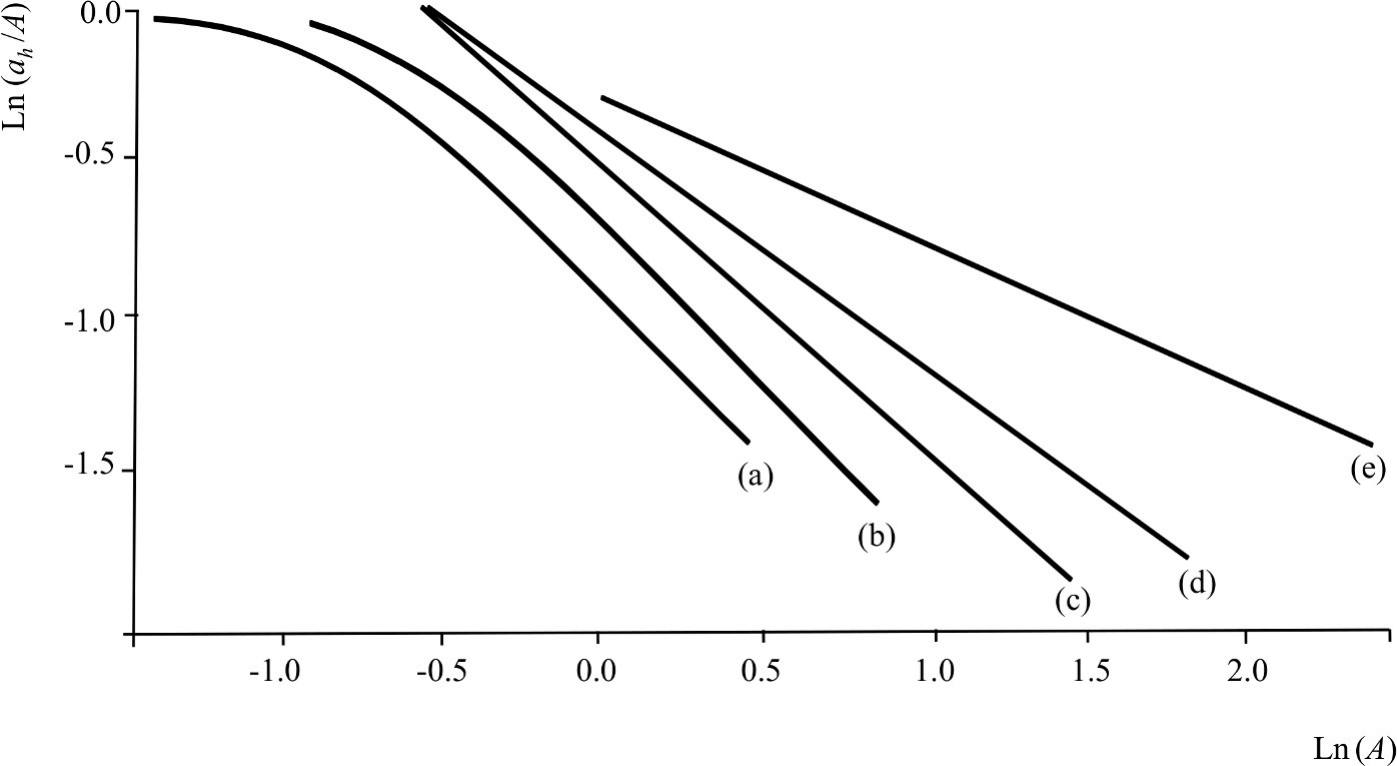
Variation in slope (*b*) influences prediction of *a*_*h*_. Prediction of *a*_*h*_ changes as *A* increases from (a,b) small cores that under-sample *a*_*h*_, through cores (c,d) with slope closer to -1 which yield the better predictions, to large cores (e) that over-sample the area covered by the animal.

**Fig 6:**
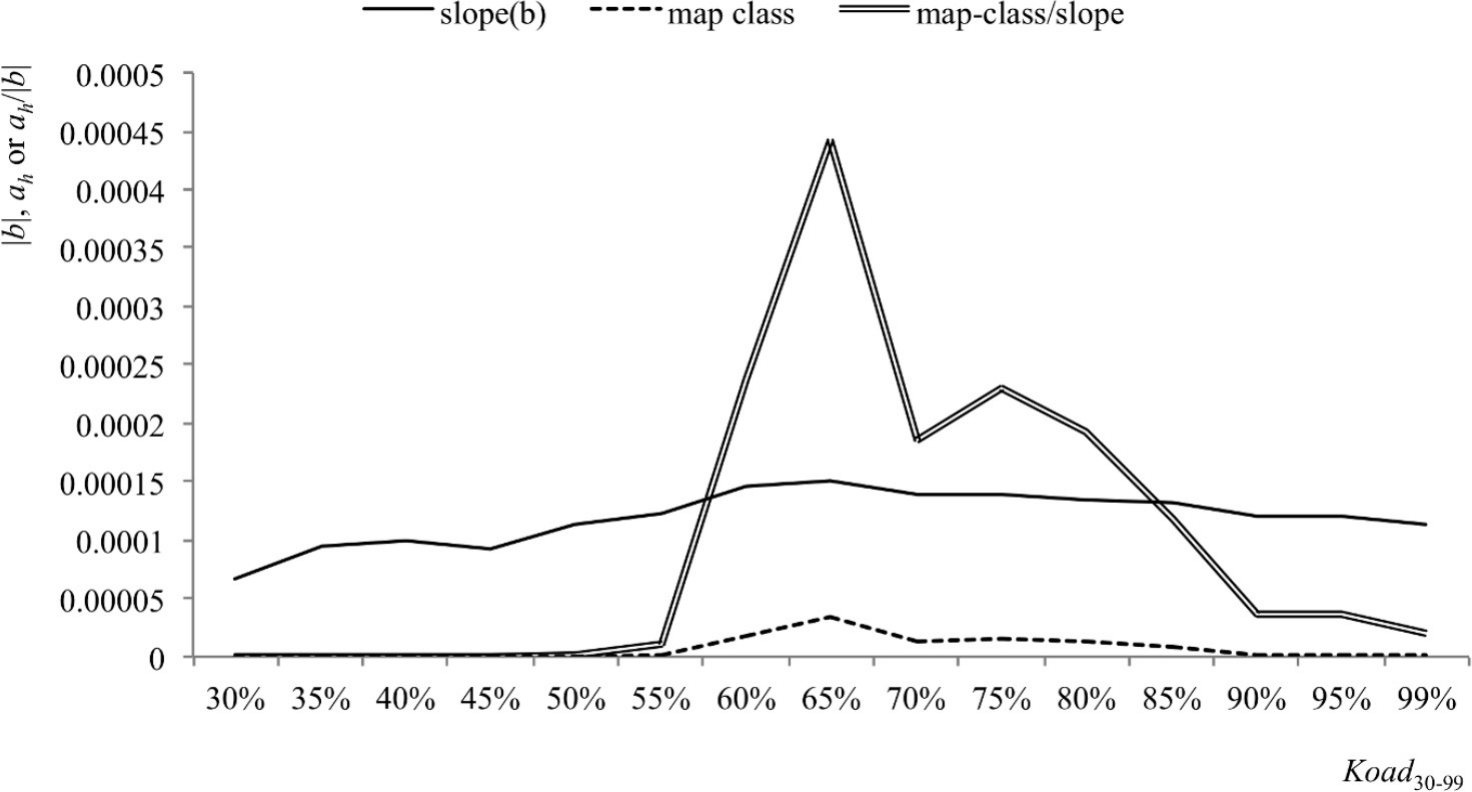
Results for buzzard home-ranges and seasonally long grass illustrate obtainment of optimal RADA prediction. Regression slope (solid line) and predicted *a*_*h*_ (dotted line) change as core size varies. The highest value for the ratio *a*_*h*_*/b* (double line) corresponds to the core at which optimal prediction is obtained; in this case, *Koad*_65_.

In general, regressions of map category area on range area for buzzards followed a pattern summarized in Fig 5. At the smallest core sizes, the occurrence of *A*_*i*_ smaller than *a*_*h*_ could produce curvilinear regressions and hence underestimation of *a*_*h*_. With increase in core size, regressions tended to become linear with steeper slopes, until a core size was reached at which range areas related less strongly to *a*_*h*_, while in larger core sizes the predicted minimum *a*_*h*_ declined. We considered that the best estimate of *a*_*h*_ was the maximum core size at which there was still a large intercept on the *x*-axis. In practice, it was convenient to assess core sizes at which *a*_*h*_ remained high relative to the absolute value of the slope (i.e. as *a*_*h*_/|*b*|), because decline in slope was more gradual owing to its logarithmic scale (Fig 6). This gave a very clear peak for *Koad*_65_, in which the meadow category had a mean area of 13.5 ha (11–16.5). None of the 114 buzzards had less than 1.4 ha of meadow within this core.

Once meadow was removed, the strongest negative RADA correlations across a range of habitats (sparse grass, open shrub, dense shrub and suburban land) were obtained for *Xr*_40_ hulls peeled to include 40% of locations nearest the range center (Table 1). When preying on small mammals, buzzards commonly hunt from and sometimes defend perches on trees along land-cover edges, such that relatively tight outlines represent an inner territorial core related to this resource. Similar strong RADA and weak placement relationships for sparse grass and the two shrub categories, plus knowledge that they all provide small mammal prey, suggested that a category formed by the union of these three could be a more meaningful representation of the distribution of the resource for buzzards. This combined map category, termed ‘rough ground’, was then in 74% of the 9.4 ha (8.1–11.0) *Xr*_40_ hulls.

**Table 1:** RADA and placement results for buzzards and nine map categories relatively well represented in Xr40 after seasonally long grass was excluded.

	RADA (missing values omitted)				Placement		
Map category	% cores	Cores	*r* obs(rand)	*P*	Cores	n% obs(rand)	*P*
**Sparse Grass**	54	35	-0.74(-0.56)	≈0.05	30–40	46(34)	<0.02
**Short Grass**	93	ns	n/a		30–100	93(66)	***<0*.*001***
**Marsh**	31	ns	n/a		35–100	53(36)	***<0*.*001***
**Open Shrub**	50	35–80	-0.67(-0.36)	**≈0.01**	ns	n/a	
**Dense Shrub**	37	30–50	-0.73(-0.36)	**<0.01**	ns	n/a	
**Deciduous Wood**	81	ns	ns		30–95	89(74)	**<0.002**
**Conifer Wood**	44	75–100	+0.41(+0.10)	<0.001	ns	n/a	
**Arable Land**	70	70–90	+0.17(-0.06)	<0.01	65–95	93(83)	**≈0.01**
**Suburban/rural**	43	30–50	-0.71(-0.26)	**<0.002**	30–55	43(58)	<0.005

The percentage of *Xr*_40_ with each map category present, spread of cores with RADA significant at *P*<0.10 and Pearson's *r* for greatest significance, followed by spread of cores and peak significance for placement of home-range cores that always contained the map category. Where RADA was not significant (ns for *P* >0.10), peak values were not applicable (n/a). Outlines in which each map category was missing were resampled. Correlations and placement results for map categories significantly underused are also shown (dotted underline). Probabilities are underlined for (*P*≤0.1), **bold** for (*P*≤0.01) or ***bold-italic*** for (*P*≤0.001).

**Fig 7:**
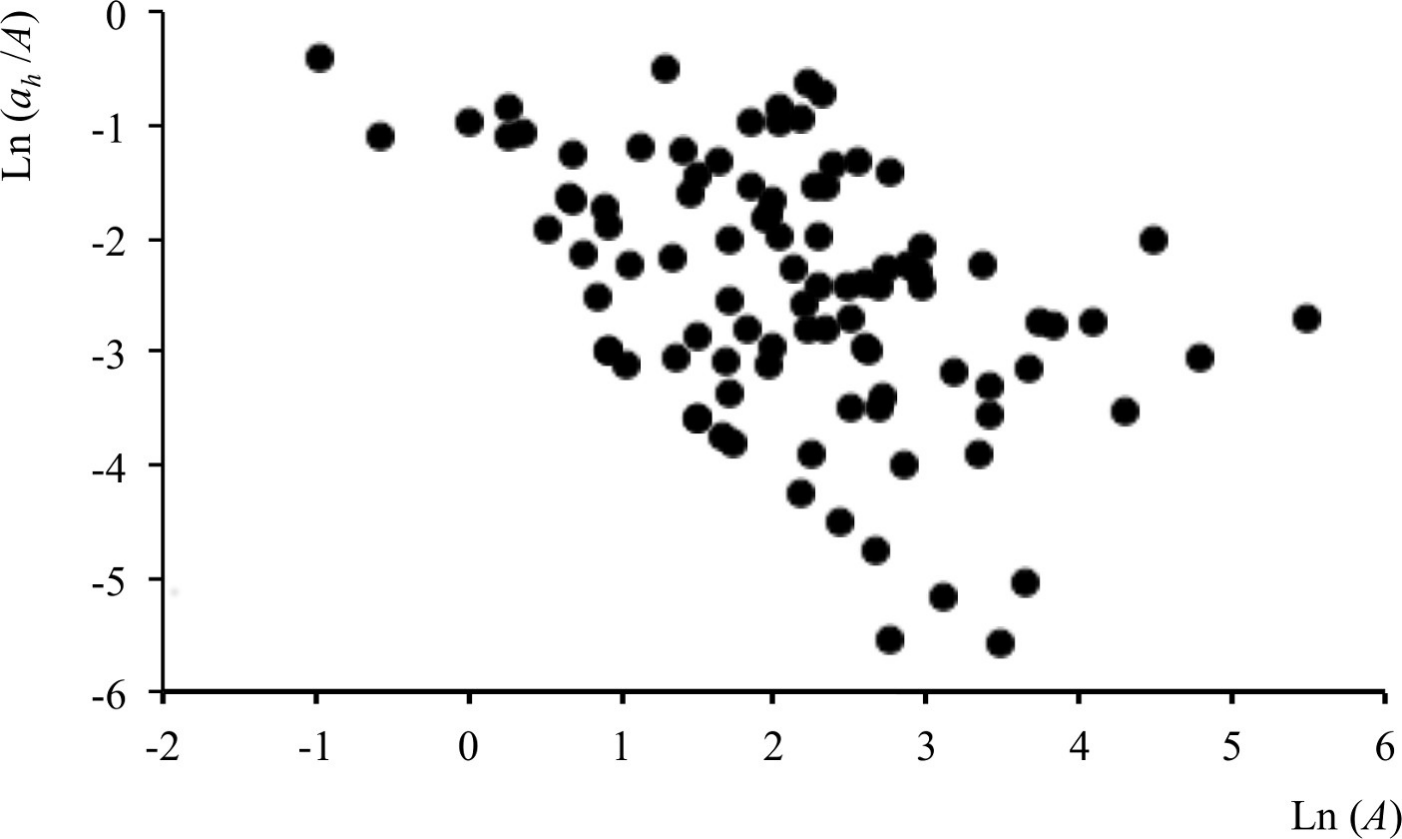
Results for buzzards and rough ground after exclusion of seasonally long grass. Proportion of rough ground (*a*_*h*_ /*A*) as a function of home-range area (*A*) after excluding seasonally long grass. *A* estimated using *Cxi*_40_. This plot also illustrates the ‘rare-pixel’ effect, which may occur when cores containing very few pixels of a resource-providing map category (cases of 1 to 3 pixels at lower left) are used in the analysis.

Repeating the analysis for the pooled-resource category considering a suite of estimators yielded even stronger correlations for *Cxi*, the convex hull including all incremental cluster nuclei. There was rough ground in 84% of the *Cxi*_40_ range outlines, and when outlines without meadow were excluded the observed strongly negative *r* = -0.45 (n = 97) differed from randomized *r* = -0.19 (95% CL -.41, +0.01, n = 999) at (P ≈ 0.01). However, the plot of this in Fig 7 indicates that a rare-pixel effect was likely to be influencing the observed and randomly generated correlations. Although the area of rough ground in the smallest range cores was equivalent to an average of 18–20 pixels, the lower edge of the distribution indicates an appreciable contribution from ranges containing a single pixel (1 case), two pixels (10) and three pixels (5). The randomly placed outlines were also affected by the rare-pixel effect of this sparsely distributed map category, so there was a negative bias to random *r* too. Replacement of missing values gave an observed *r* = -0.25, while in this case also tending to give positive *r* values by randomization, with mean +0.15 (95% CL -0.07,+0.33, n = 999) and no random *r* values less than -0.25 in two runs (*P* < 0.001).

Plotting *a*_*h*_/|*b*| for *Cxi* gave *Cxi*_35_ as the best estimator for *a*_*h*_. However, the area prediction of 0.09 ha (0–0.25) was considerably smaller than ${\bar{a}}_{h}$, the mean 0.54 ha (0.35–0.82) area of rough ground associated with this core. Here, therefore, the mean again seemed a better predictor of the buzzards’ rough ground requirements.

In the same *Cxi*_35,_ after long grass and rough ground were excluded, the suburban map category–almost exclusively gardens–gave *r* = -0.52 with randomized *r* = -0.21 (95% CL -.42, +0.01, n = 999) and 2 random *r* values less than -0.52 (*P* < 0.005) and mean area requirement estimate (${\bar{a}}_{h}$) of 0.41 ha (0.29–0.59). This category was present in 51% of *Cxi*_35_ ranges, indicating the existence of divergent hunting strategies among tagged buzzards.

## Discussion

**Fig 8:**
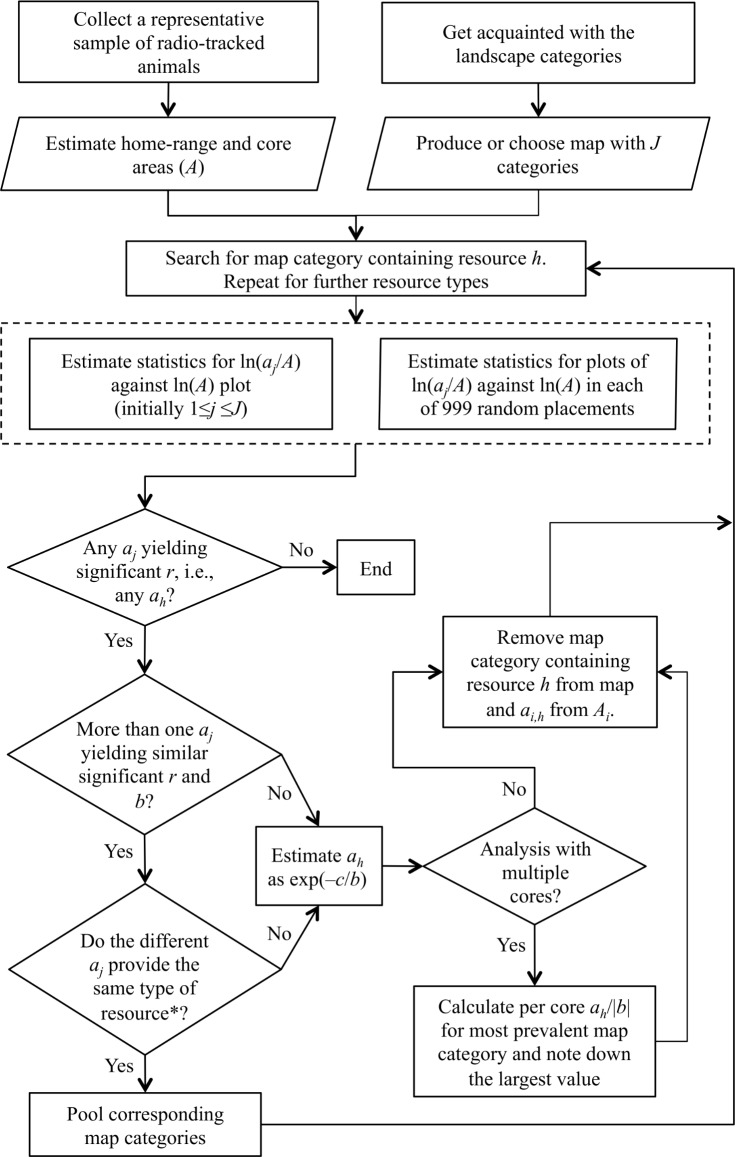
An implementation of Resource-Area-Dependence Analysis. Given *J* map categories, denote the area of a map category *j* contained within *A*_*i*_ by *a*_*i*,*j*_, where *A*_*i*_ denotes the home-range area of animal *i* (for a chosen home-range estimator). Using the regression of ln(*a*_*j*_/*A*) against ln(*A*) with the most significant *r* (tested by randomization), estimate *a*_*h*_, the minimum area requirement of the map category providing resource *h*, from the slope *b* and intercept *c*. Repeat, removing the identified map category, until no further map category yields significant results. *Clarifying this requires ancillary information, such as from fieldwork or literature.

RADA makes it possible to determine which map category contains an important accessible resource, which home-range core the required resource amount is associated with, and what area of the resource-containing map category is required on average by individuals. We have illustrated this by applying it to two species of squirrels and a buzzard, as shown in Fig 8 for the implementation defined in methods with further refinement developed in the results.

RADA differs from other habitat ‘use-availability’ analyses in three important ways. First, it can extend beyond relative preference analysis, which compares use with availability at one or two discrete spatial scales, by considering relationships over a series of home-range core sizes. Second, unlike previous analyses, RADA does not treat every map category (or ‘habitat’) within a home-range core as ‘used’, which can bias results when there are unselected or unused map categories [34]. Third, RADA provides estimates of individual resource requirements, which may be convenient as input for studying landscape suitability with agent-based modeling.

### Home-range-based interpretation

Results were significant for map categories that were known from independent observations to contain important resources for each species. Squirrels require woodland for seeds and nests [47,55], although gray squirrels also take foods such as wheat in summer [48]. RADA showed dependence of red squirrel range size on mature woodland (Fig 3) and more strongly significant relationships between gray squirrel ranges and wheat (Fig 4) than in either of two previous analyses that used the same data set to examine habitat preferences [22,25]. For buzzards, too, seasonally long grass and rough ground categories not only correlated with range size (Table 1 and Fig 7), but also affected productivity and persistence in an area [52,56]. Arable land, which was favored in extensive outlines, but underused in cores (Table 1), has been recognized as a ‘poison chalice’ category associated with dispersal [52].

The closer the slope *b* is to -1, the more precise the area estimates are. A strong relationship of this nature indicated resource-area-dependence for gray squirrels at Elton, who required 0.35–0.46 ha of wheat in summer.

However, shallower negative slopes lead to underestimation of *a*_*h*_. Results for buzzards illustrate this point. No buzzards had as little meadow in their *Koad*_65_ as the 0.05 ha upper confidence limit for the regression, and fewer than 5% had as little rough ground as the 0.08 ha predicted by regression for the *Cxi*_35_. In cases such as these, where a lower 95% CL for observed values does not overlap an upper 95% CL for the intercept, an alternative is to consider the mean area of the *h*-containing map category in the outline for which there is a strong relationship, knowing that this may over-estimate the average animal’s minimum resource requirements. For buzzards, this yielded 14 ha (11–17) of meadow and 0.54 ha (0.35–0.82) of rough ground. Similarly, for red squirrels on Furzey Island of 0.48 ha (0.24–0.97) ha of pine woodland and for gray squirrels at Elton of 0.34 ha (0.11–1.12) of deciduous woodland. These mean areas of woodland were appropriate for non-overlapping cores of females, which were at densities of 2/ha on Furzey Island and 1.5/ha at Elton [47,57].

Where resource accessibility across map categories is comparable, similar RADA results are expected even if the map categories are analyzed separately. This was likely the case for buzzards hunting small mammals [56] in the sparse grass and the two shrub categories, which yielded very similar results. In such cases, it makes sense to group categories in a way that is more biologically meaningful to the animal and redo the analysis, as was done here by combining the sparse grass and the shrub categories into the rough ground category that then yielded even stronger results (Table 1).

In this exploration using ranges estimated from VHF telemetry datasets, we used 25 estimators, including those in an earlier review [46] and with addition of a Local Convex Hull (LoCoH) analogue. In order to minimize risk of Type I errors, one solution is to investigate no more than five estimators and reduce the level of probability considered to be statistically significant from *P* < 0.05 to *P* < 0.01. From experience here and previously, we would recommend (i) ellipse (*Ejt*) and (ii) kernel (*K10d* or *Koad*) as expansive, density-based estimators, with (iii) peeled hull *Xr* (which includes the outer MCP as *Xr*_100_) and separate nearest-neighbour cluster (iv) *Cxs* as link-distance estimators for tight cores, especially on granular maps. If (iii) and (iv) give strong relationships, then the single hull round cluster nuclei *Cxi* is a good choice for (v), but if only (iv) is strong then a further option with separate clusters or concavity (*ix* or *Cv* or a LoCoH analogue) is indicated as (v).

### Animal population considerations

A tacit assumption of RADA is that animals are obliged by energy considerations or competition to have the resources they need within their home-ranges, even if overlap of more excursive areas is not strictly ‘area-minimizing’ [58]. Otherwise, relationships between home-range area and resource content may be weak.

There may also be divergent strategies with regards to resource use by the animals under study [59]. When this can be discovered from *a priori* evidence (e.g. [8]), or is suggested from the log-log plot, statistical separation of distributions for each specialist group (e.g. [60]) may justify separate analyses. Combination of range placement analysis with RADA may also be important for detecting divergence. In buzzards, compact range outlines were placed away from the suburban map category for a significant proportion of birds (Table 1), but the proportion of birds (about half) that had this map category within small cluster-based hulls tended to show area-dependence.

### Robust application

For RADA, the quality of results is intimately associated with how well the biological pattern was detected by the datasets and the model assumptions were met. For example, variation in resource homogeneity within patches will militate against detecting resource importance. Significant *b* values for seasonally long grass of around -0.2 in buzzard ranges (as opposed to being closer to -1.0) were probably due to this map category in the LCMGB not representing homogeneously distributed resources. Buzzards will have been feeding on earthworms [56] and it is plausible that larger patches in larger ranges may have represented cultivated grassland, where worm densities are lower than in small permanent paddocks. Resources such as worms will have been available in most other map categories, but were most accessible to or desired by buzzards in seasons when grass was short. In future analyses, we suggest that the map category ‘seasonally long grass’ be split into ‘cultivated grassland’ and ‘paddocks’ (<1ha).

Fortunately, the ability to collect better data is advancing fast owing to developments in remote sensing and animal tracking. It is, for example, nowadays possible to map at different spatial or temporal resolutions land- or vegetation-cover [61,62], species composition [63], flooding gradient [64–66] or the entire three-dimensional vegetational structure [67]. This allows homing into mapping resources for animals that live in open areas, under or within canopies or in places with complicated relief. Image processing algorithms have also improved. For example, spatial resolution can be increased by fusing spectral bands with the panchromatic band, or classification results can be optimized by confronting results from a suite of automated or supervised classification methods [68–70]. Importantly for ecologists seeking long term data, there is a growing body of techniques for taking advantage of most, if not all, images from temporal series of satellite imagery, such as that from the Landsat sensors [71,72]. In summary, this field is advancing fast and with it the ability to map resources.

Technologies for assessing animal movement, on their side, have also been undergoing major advances, ushered in initially by Very High Frequency (VHF) technology and more recently by Global Positioning Systems (GPS) [31,53,73,74], with emerging low-cost options increasing the accessibility of researchers and conservationists to this technology [75]. Current algorithms make better use of the added information provided by GPS data, leading to more refined home-range estimates [76–78] or predictions [58,79–82]. Moreover, better ways of estimating *a*_*h*_ than our proposed ‘maximum core size at which there was still a large intercept on the *x*-axis’ may emerge from more extensive use of RADA. The concept of home-range itself, for which no universal definition has been agreed upon, has also been maturing [53,74,83–85]. Such advancements will allow for increased objectivity when choosing the estimator for use in RADA, which will, in turn, contribute to reducing the likelihood of Type I errors.

Having said this, from the perspective of RADA as a method it is encouraging that clear patterns and quite strong relationships (Figs 3, 4 and 7) were obtained with maps produced with 30-year-old imagery and samples for home-range estimation considerably smaller than the usual GPS samples of today. Better datasets and concepts should lead to more robust RADA results about how animals’ home-ranges are structured by resources.

## Supporting information

S1 Appendix Details and abbreviations for the 25 home-range variants used in analyses and their cores (subscripts refer to core sizes). (DOCX)

S1 Table Comparison of coefficients for correlations for red squirrels and pine woodland. (DOCX)

S1 Fig Obtaining optimal RADA prediction for buzzards and rough ground. (TIFF)
