# Supplementary material for: Resource-Area-Dependence Analysis: Inferring animal resource needs from home-range and mapping data
Source: PLoS One. 2018 Oct 24;13(10):e0206354. doi: 10.1371/journal.pone.0206354 (PMC6200262; doi:10.1371/journal.pone.0206354)
Supplement: S1 Appendix — (DOCX) [file pone.0206354.s001.docx]

**S1 APPENDIX**

Definitions of abbreviations used for home range outline estimators. Subscripts refer to core sizes.

Abbreviation Description___________________ _____________________________

Location-density isopleths:

*Ejt30-99* Ellipse, Jennrich-Taylor type

*K4d30-99-K12d30-99* Kernel isopleth, 40x40 grid, 0.4x-1.2x standard smoothing, density-inclusive

*Kod30-99* Kernel, 40x40 grid, LSCV-optimized smoothing, density-inclusive

*Koad30-99* Kernel, 40x40 grid, LSCV-optimized smoothing, adaptive, density-inclusive

*Koal30-100* Kernel, 40x40 grid, LSCV-optimized smoothing, adaptive, location-inclusive

*Hud30-99* Harmonic mean isopleth, 150x150 grid, uncentred locations, density-inclusive

*Hul30-100* Harmonic mean isopleth, 150x150 grid, uncentred locations, location-inclusive

Peeling of peripheral locations or incremental cluster analysis defines hulls or concave polygons:

*Xk30-100* Convex hull, peeled by distance from kernel range center

*Xr30-100* Convex hull, peeled by distance from recalculated arithmetic mean

*Cxi30-100* Cluster analysis, one convex hull includes all clusters

*Cxs30-100* Cluster analysis, separate convex hull round each cluster

*Cv30-100* Cluster analysis, polygons round locations with half-span edge-restriction per cluster

One point on the distribution of nearest neighbor distances limits internal cluster distances:

*tx05* Truncation of outer 5% of nearest neighbor distance distribution, convex hull

*ix01* (*ix001*) Iterative exclusion of outer 1% (0.1%) of distance distribution, convex hulls

*tv05* Truncation, polygons round locations with half-span edge-restriction

*iv01* (*iv001*) Iterative exclusion of n-n distances, polygons with half-span edge-restriction

One point on the distribution of nearest neighbor distances limits edge lengths:

*ot05* Truncation of nearest neighbor distances sets hull and hole edge lengths

*oi01* (*oi001*) Iterative exclusion of n-n distance distribution sets hull and hole edge lengths

______________________________
