# Supplementary material for: Resource-Area-Dependence Analysis: Inferring animal resource needs from home-range and mapping data
Source: PLoS One. 2018 Oct 24;13(10):e0206354. doi: 10.1371/journal.pone.0206354 (PMC6200262; doi:10.1371/journal.pone.0206354)
Supplement: S1 Table — (DOCX) [file pone.0206354.s002.docx]

|  | **Percentage inclusion of density distribution or locations** | | | | | | | | | | | | | | |
| --- | --- | --- | --- | --- | --- | --- | --- | --- | --- | --- | --- | --- | --- | --- | --- |
|  | **30%** | **35%** | **40%** | **45%** | **50%** | **55%** | **60%** | **65%** | **70%** | **75%** | **80%** | **85%** | **90%** | **95%** | **99/100%** |
| ***Ejt*** | -0.36 | -0.34 | -0.34 | -0.35 | -0.37 | -0.40 | -0.44 | -0.49 | ***-0.54*** | ***-0.61*** | ***-0.67*** | ***-0.73*** | ***-0.78*** | ***-0.84*** | ***-0.87*** |
| ***K4d*** | 0.06 | -0.02 | -0.15 | -0.21 | -0.25 | -0.28 | -0.30 | -0.34 | -0.37 | -0.40 | -0.44 | -0.49 | ***-0.53*** | ***-0.61*** | ***-0.68*** |
| ***K6d*** | -0.08 | -0.11 | -0.15 | -0.20 | -0.26 | -0.34 | -0.41 | -0.48 | ***-0.53*** | ***-0.56*** | ***-0.60*** | ***-0.65*** | ***-0.71*** | ***-0.78*** | ***-0.82*** |
| ***K8d*** | -0.12 | -0.20 | -0.27 | -0.32 | -0.36 | -0.40 | -0.45 | ***-0.53*** | ***-0.58*** | ***-0.63*** | ***-0.68*** | ***-0.74*** | ***-0.79*** | ***-0.83*** | ***-0.86*** |
| ***K10d*** | -0.21 | -0.28 | -0.33 | -0.37 | -0.43 | -0.48 | -0.51 | ***-0.55*** | ***-0.62*** | ***-0.67*** | ***-0.73*** | ***-0.78*** | ***-0.82*** | ***-0.84*** | ***-0.85*** |
| ***K12d*** | -0.25 | -0.32 | -0.36 | -0.42 | -0.47 | -0.51 | ***-0.54*** | ***-0.58*** | ***-0.65*** | ***-0.72*** | ***-0.77*** | ***-0.80*** | ***-0.81*** | ***-0.83*** | ***-0.87*** |
| ***Kod*** | -0.34 | -0.41 | -0.46 | -0.49 | -0.49 | -0.49 | -0.50 | ***-0.53*** | ***-0.58*** | ***-0.61*** | ***-0.64*** | ***-0.67*** | ***-0.69*** | ***-0.72*** | ***-0.68*** |
| ***Koad*** | -0.18 | -0.26 | -0.33 | -0.39 | -0.43 | -0.48 | ***-0.53*** | ***-0.58*** | ***-0.66*** | ***-0.73*** | ***-0.78*** | ***-0.82*** | ***-0.85*** | ***-0.84*** | ***-0.89*** |
| ***Koal*** | 0.11 | 0.16 | -0.09 | -0.08 | -0.21 | -0.28 | -0.43 | -0.44 | ***-0.52*** | ***-0.66*** | ***-0.76*** | ***-0.73*** | ***-0.82*** | ***-0.86*** | ***-0.90*** |
| ***Hud*** | 0.08 | 0.16 | 0.18 | 0.14 | 0.07 | -0.01 | -0.16 | -0.23 | -0.31 | -0.38 | -0.45 | -0.50 | ***-0.54*** | ***-0.55*** | ***-0.59*** |
| ***Hul*** | -0.01 | 0.10 | 0.06 | -0.03 | -0.05 | -0.21 | -0.22 | -0.19 | -0.28 | -0.13 | -0.44 | -0.44 | ***-0.63*** | ***-0.51*** | ***-0.64*** |
| ***Xk*** | 0.13 | 0.04 | 0.05 | 0.09 | -0.15 | -0.23 | -0.24 | -0.25 | -0.26 | -0.29 | -0.38 | -0.32 | ***-0.50*** | -0.26 | -0.25 |
| ***Xr*** | 0.13 | 0.14 | -0.08 | -0.05 | -0.10 | -0.12 | -0.26 | -0.27 | -0.32 | -0.30 | ***-0.52*** | -0.41 | ***-0.56*** | -0.25 | -0.25 |
| ***Cxi*** | -0.22 | -0.24 | -0.30 | -0.30 | -0.45 | ***-0.53*** | -0.39 | -0.42 | -0.32 | -0.29 | ***-0.55*** | ***-0.55*** | ***-0.57*** | -0.04 | -0.25 |
| ***Cxs*** | -0.14 | -0.13 | -0.15 | -0.08 | -0.01 | 0.02 | -0.18 | -0.20 | -0.41 | -0.42 | -0.46 | ***-0.63*** | ***-0.67*** | ***-0.54*** | ***-0.69*** |
| ***Cv*** | -0.16 | -0.11 | -0.13 | -0.05 | 0.01 | 0.02 | -0.22 | -0.21 | -0.46 | -0.46 | -0.44 | -0.49 | ***-0.58*** | ***-0.61*** | ***-0.61*** |
|  |  |  |  |  |  |  |  |  |  |  |  |  |  |  |  |
|  | **tx05** | **tv05** |  |  | **ix01** | **iv01** |  |  | **ix001** | **iv001** |  |  | **ot05** | **oi01** | **oi001** |
| **88%** | -0.27 | -0.29 |  | **87%** | ***-0.74*** | ***-0.60*** |  | **94%** | ***-0.86*** | ***-0.68*** |  | **OREP** | ***-0.52*** | ***-0.67*** | -0.25 |
