# Supplementary figures and images for: Resource-Area-Dependence Analysis: Inferring animal resource needs from home-range and mapping data
Source: PLoS One. 2018 Oct 24;13(10):e0206354. doi: 10.1371/journal.pone.0206354 (PMC6200262; doi:10.1371/journal.pone.0206354)

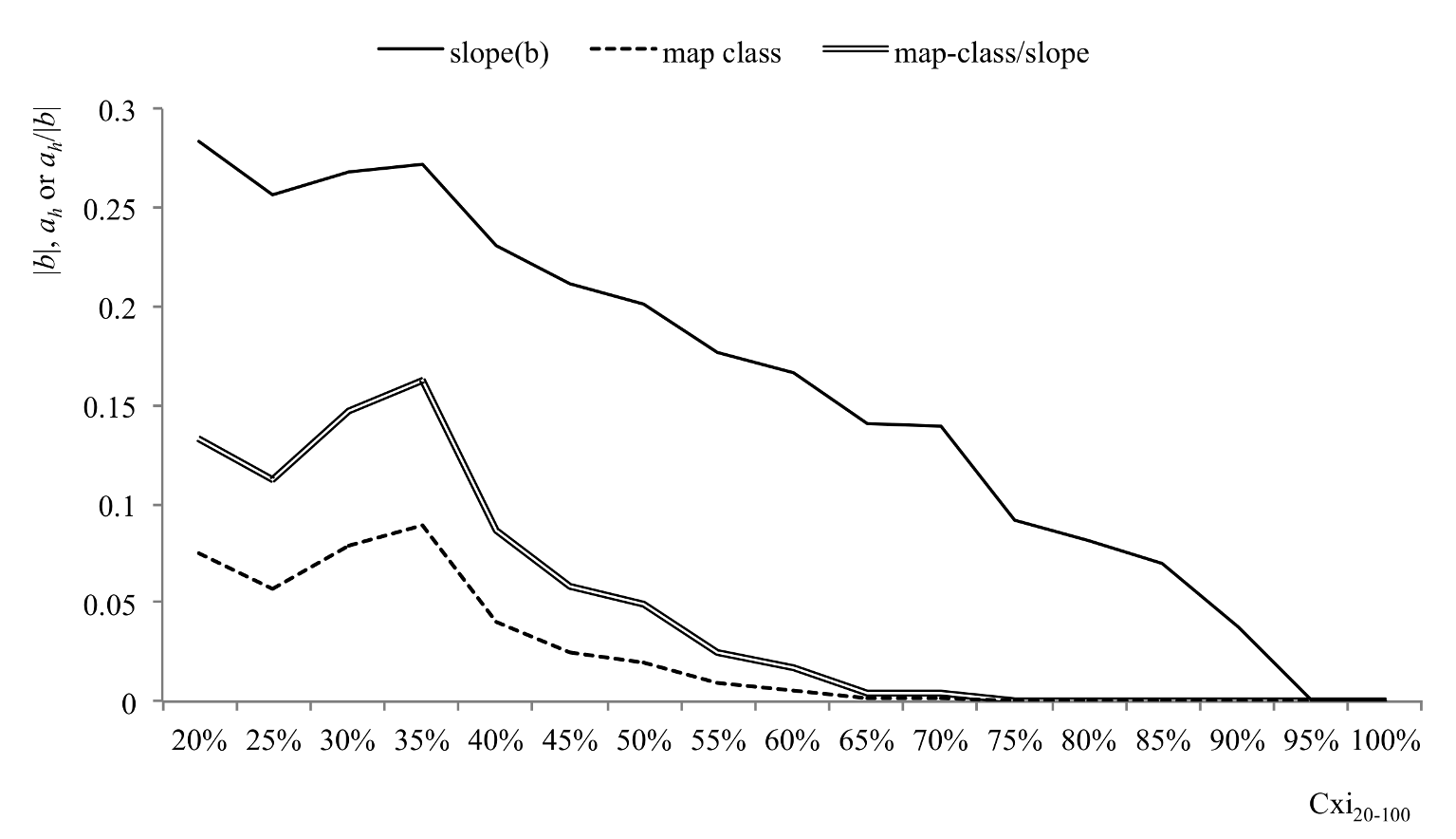

Supplement: S1 Fig — (TIFF) [file pone.0206354.s003.tiff]
